# Development of an Intervention Targeting Multiple Health Behaviors Among High School Students: Participatory Design Study Using Heuristic Evaluation and Usability Testing

**DOI:** 10.2196/17999

**Published:** 2020-10-29

**Authors:** Ulrika Müssener, Kristin Thomas, Catharina Linderoth, Marie Löf, Katarina Åsberg, Pontus Henriksson, Marcus Bendtsen

**Affiliations:** 1 Department of Health, Medicine and Caring Sciences Linköping University Linköping Sweden; 2 Department of Biosciences and Nutrition Karolinska Institutet Huddinge Sweden

**Keywords:** mHealth intervention, health behavior, high school students, participatory design, heuristic evaluation, usability testing, mobile phone

## Abstract

**Background:**

Mobile electronic platforms provide exciting possibilities for health behavior promotion. For instance, they can promote smoking cessation, moderate alcohol consumption, healthy eating, and physical activity. Young adults in Sweden are proficient in the use of technology, having been exposed to computers, smartphones, and the internet from an early age. However, with the high availability of mobile health (mHealth) interventions of varying quality, it is critical to optimize the usability of mHealth interventions to ensure long-term use of these health promotion interventions.

**Objective:**

This study aims to investigate the usability of an mHealth intervention (LIFE4YOUth) targeting health behaviors among high school students through heuristic evaluation and usability testing.

**Methods:**

A preliminary version of the LIFE4YOUth mHealth intervention, which was aimed at promoting healthy eating, physical activity, smoking cessation, and nonrisky drinking among high school students, was developed in early 2019. We completed a total of 15 heuristic evaluations and 5 usability tests to evaluate the usability of the mHealth intervention prototype to improve its functioning, content, and design.

**Results:**

Heuristic evaluation from a total of 15 experts (10 employees and 5 university students, both women and men, aged 18-25 years) revealed that the major usability problems and the worst ratings, a total of 17 problems termed *usability catastrophes*, concerned shortcomings in displaying easy-to-understand information to the users or technical errors. The results of the usability testing including 5 high school students (both girls and boys, aged 15-18 years) showed that the design, quality, and quantity of content in the intervention may impact the users’ level of engagement. Poor functionality was considered a major barrier to usability. Of the 5 participants, one rated the LIFE4YOUth intervention as poor, 2 rated as average, and 2 assessed it as good, according to the System Usability Scale.

**Conclusions:**

High school students have high expectations of digital products. If an mHealth intervention does not offer optimal functions, they may cease to use it. Optimizing the usability of mHealth interventions is a critical step in the development process. Heuristic evaluation and usability testing in this study provided valuable knowledge about the prototype from a user’s perspective. The findings may lead to the development of similar interventions targeting the high school population.

## Introduction

### Health Behaviors Among Young People

Chronic diseases are the leading cause of death and disability worldwide. Globally, up to an estimated 80% of cases of heart disease, stroke, and type 2 diabetes and more than 30% of cancers can be prevented by reducing smoking, harmful alcohol use, improving diet, and engaging in regular physical activity [1,2]. Previous research has shown that multiple risk behaviors increase the risk of chronic disease and all-cause mortality, more so than the combined effects of single behaviors [3-5]. Health behaviors typically emerge during adolescence, track into adulthood, and commonly co-occur [6-8]. Therefore, adolescence is a critical age to intervene and interrupt a trajectory toward poor adult health [9-11]. Evidently, effective and evidence-based health behavior promotion interventions are needed.

### Health Behavior Promotion Among Youths Through Multiple Mobile Health Interventions

Mobile platforms provide exciting possibilities for the promotion of health behaviors through mobile health (mHealth) interventions. Previous research has shown that interventions targeting multiple health behaviors at the same time might be effective in improving the general lifestyle among adults [5,12], with less evidence among adolescents [13]. A meta-analysis [14] examined the effectiveness of text message–based interventions for tobacco and alcohol cessation in a young adult population. Only 5 of the 14 studies reported significant differences between groups of substance use behavior outcomes, and the included randomized controlled trials (RCTs) lacked details regarding the intervention content. A more recent systematic review and meta-analysis [15] investigated the effectiveness of school-based eHealth interventions, defined as interventions delivered via the internet, computers, mobile technology, or telehealth, to prevent multiple health risk behaviors among adolescents. A total of 22 publications were included, assessing 16 interventions that targeted 2 or more of the following behaviors: alcohol use, smoking, diet, physical activity, screen time and sitting, or sleep. Only short-term effects were found for improving physical activity, screen time, and fruit and vegetable intake, and all trials were considered to be of low quality. There was limited evidence of their effect on alcohol and smoking. Although the selection criteria in the meta-analysis included studies with intervention components delivered via mobile technology, no studies of mHealth-only interventions were identified [15].

### The Development of mHealth Interventions

Young adults are referred to as digital natives; many are proficient in the use of technology, having been exposed to computers, smartphones, and the internet from an early age. Indeed, young adults have the highest level of smartphone ownership among all age groups [16]. It is critical to optimize the usability of interventions targeting this age group; if they do not enjoy the program, they may disengage. Several reviews have shown the feasibility, acceptability, and efficacy of digital interventions for behavior change [17,18] and other health interventions, such as disease self-management [19-21] and medication adherence [22,23]. Previous systematic reviews on texting and mobile app interventions emphasized the urgent need to examine the development processes of mHealth interventions [23,24].

In a participatory design approach, research is undertaken with, rather than on, people, allowing researchers to gain an understanding of context-specific requirements and challenges [25]. Heuristic evaluation and usability testing are commonly used to support the development and refinement of the effectiveness of mHealth interventions. During heuristic evaluation, trained evaluators review an intervention to find usability problems, assign them to a specific category of heuristics, and ascribe a severity rating to provide distinct usability information [26,27]. Heuristics are often used to identify usability issues, such as problems with unclear functions, confusing navigation, and consistency issues [27-29]. Usability tests consist of a human-computer interaction and refer to evaluating an intervention by testing it with potential end users with the goal of identifying understandability, ease of learning, and attractiveness and to determine participants’ satisfaction with the intervention. Usability testing provides developers with feedback about what does and does not work in the intervention and determines whether the features in the interventions are acceptable to and feasible for users and also determines what can be improved [30-33].

### Objectives

This study aims to investigate the usability of an mHealth intervention (LIFE4YOUth) targeting health risk behaviors among high school students through heuristic evaluation and usability testing.

## Methods

### Procedures

A preliminary version of the LIFE4YOUth mHealth intervention targeting health risk behaviors among high school students was developed in early 2019. LIFE4YOUth is one of 7 multiple mHealth interventions in the MoBILE (mHealth–Multiple Lifestyle Behaviors) research program (funded by Forte 2018-01410, principal investigator: ML) aimed at promoting healthy eating, physical activity, smoking cessation, and nonrisky drinking in 7 different populations in the health care system. The intervention includes information about health behaviors, tips on behavior change strategies, and activities. The formative research process of developing a novel multiple mHealth intervention is described in detail in a study protocol elsewhere [34]. This study reports on findings from the first stage of the formative research process: heuristic evaluation and usability testing. The aim is to investigate the usability of a prototype app in terms of function, content, and design.

### Setting, Participants, and Recruitment

#### Heuristic Evaluation

Recruitment of participants for the heuristic evaluation was undertaken by members of the research team through paper advertisements (posters) in public areas at Linköping University, Sweden. The inclusion criteria for the heuristic evaluation were university students and employees, both women and men, aged 18 to 25 years, at the Faculty of Medicine and Health Sciences at Linköping University who were willing to participate and owned a mobile phone. Participants showed their interest by contacting the research leader via email or telephone.

#### Usability Tests

School staff at 5 high schools selected for convenience in Östergötland, Sweden, were contacted via email and informed about the research project. Students from all schools were invited to participate in the usability testing. The recruitment of participants was performed by school staff through paper advertisements (posters and leaflets), digital advertisements (student email and school website), and information disseminated in the classrooms. The inclusion criteria for the usability tests were high school students, both female and male, aged 15 to 18 years, willing to participate and owning a mobile phone. High school students showed their interest by contacting the research leader via email or telephone.

### Data Collection

#### Informed Consent

All participants provided written informed consent before participation in all study procedures (heuristic evaluation and usability tests).

#### Heuristic Evaluations

A total of 15 experts (10 employees and 5 university students) were recruited. For the heuristic evaluation, a set of 10 heuristics published by Nielsen [26] was used to evaluate the LIFE4YOUth prototype. The heuristics for usability evaluation according to Nielsen are listed in Textbox 1.

We selected Nielsen’s 10 heuristics because they have been thoroughly tested and are quick and easy to apply. When applying heuristic evaluation, participants evaluate an app to find usability problems, assign them to a specific category of heuristics, and assign a severity rating [26,27]. All participants were invited to a brief training session (approximately 45 min) conducted by a research assistant (CL), to receive instructions on the main principles of heuristic evaluation, and to learn to use the heuristics to evaluate the intervention [27]. The participants were sent a link to a high-fidelity prototype [35] of the intervention, including the actual software start page, menu page, and the 4 intervention modules (alcohol, smoking, physical activity, and diet). Each participant was asked to identify usability problems independently in a given protocol (Multimedia Appendix 1). Participants were asked to identify a problem, describe it, identify the relevant heuristic for the problem (eg, visibility of system status or match between the system and the real world), and give it a severity rating [26,27,29].

The procedure itself was a two-part process: the participants first familiarized themselves with the system and its usage with reference to the materials and training provided by the assistant. Then, they independently applied the 10 heuristics, as given in Textbox 1. The participants detected a usability problem, assigned each problem to a violation of a heuristic, and described the problem in their own words. Then, participants assigned severity scores to each problem by using the severity rating factors of impact presented in Textbox 2.

The introduction took place in the beginning of May 2019 in a conference room at Linköping University, Sweden. The evaluations were performed wherever the participants preferred and were sent to the research assistant in a prepaid envelope. After 10 days, a reminder was sent by a text message. Heuristic evaluations from all participants (n=15) were gathered during the last week of May 2019.

**Heuristics for usability evaluation according to Nielsen.**

1. Visibility of system status: The system should always keep users informed about what is going on through appropriate feedback within reasonable time
2. Match between system and the real world: The system should speak the users’ language, with words, phrases, and concepts familiar to the user, rather than system-oriented terms. Follow real-world conventions, making information appear in a natural and logical order
3. User control and freedom: Users often choose system functions by mistake and will need a clearly marked *emergency exit* to leave the unwanted state without having to go through an extended dialog. Support undo and redo
4. Consistency and standards: Users should not have to wonder whether different words, situations, or actions mean the same thing
5. Error prevention: Even better than good error messages is a careful design, which prevents a problem from occurring in the first place. Either eliminate error-prone conditions or check for them and present users with a confirmation option before they commit to the action
6. Recognition rather than recall: Minimize users’ memory load by making objects, actions, and options visible. The user should not have to remember information from one part of the dialog to another. Instructions for the use of the system should be visible or easily retrievable whenever appropriate
7. Flexibility and efficiency of use: Accelerators—unseen by the novice user—may often speed up the interaction for the expert user such that the system can cater to both inexperienced and experienced users. Allow users to tailor frequent actions
8. Aesthetic and minimalist design: Dialogs should not contain information that is irrelevant or rarely needed. Every extra unit of information in a dialog competes with the relevant units of information and diminishes their relative visibility
9. Help users recognize, diagnose, and recover from errors: Error messages should be expressed in plain language (no codes), precisely indicate the problem, and constructively suggest a solution
10. Help and documentation: Although it is better if the system can be used without documentation, it may be necessary to provide help and documentation. Any such information should be easy to search, focused on the user’s task, list concrete steps to be carried out, and not be too large

**Scale for severity rating according to Nielsen.**

0: Not a usability problem

1: Cosmetic problem only—need not to be fixed unless extra time is available

2: Minor usability problem—fixing this should be given low priority

3: Major usability problem—important to fix, should be given high priority

4: Usability catastrophe—imperative to fix this before product can be released

#### Usability Tests

The usability test consisted of a human-computer interaction evaluation, which focused on the perceptions and performance of users in a laboratory setting. It entailed the completion of 30 goal-oriented tasks by targeted end users [30,32]. In total, 5 usability tests were completed with high school students. The tests were carried out in June 2019 in a medical informatics laboratory room and were run on an iPhone. The participants went through a 60-min session, during which all interactions with the intervention were recorded using video. The same prototype, as in the heuristic evaluation, was used. The test manager gave the participants printouts with the 30 tasks. Examples of tasks used in the usability testing are described in Textbox 3.

The participants were asked to complete the 30 tasks, and the test manager asked the participants to explain their actions as they performed them using a think-aloud method [36,37]. An observer (the research leader) noted potential issues as the given tasks were performed by the participants. After completing the session, the participants completed a paper version of the System Usability Scale (SUS), providing a global view of their subjective assessment of usability based on 10 questions [38]. SUS has been shown to be reliable and valid with a variety of different technologies and users.

**Examples of tasks in the usability testing.**

- Go to the start page and explain what you think of the layout
- Where can you find support to drink less?
- Log what you drink. Explain how you interpret the graphs for the alcohol consumption you logged
- You know that people feel healthier after exercising and want to find out more about getting started. How would you look for this information?
- If you want to plan a physical activity, how do you find that activity, and specify your level of involvement?
- You can set personal goals for your eating habits under the “Timeline” tab. Explain how to set your goal and what the graph shows you
- What information is there under the “Risks” tab in the Diet module? Does it give you a good overview?
- Go to the Smoking module. What do you think about the scope of the information provided on the first page?
- If you want to know more about the benefits of quitting smoking, how would you go about this?

### Data Analyses

#### Heuristic Evaluation

The 10 heuristics were pooled, and the identified problems were categorized as major issues [26-29]. The focus of the analysis was to identify usability problems and critical issues and to explore whether any functions in the intervention performed better than others. A master list was compiled to collect all the described usability problems, duplicate problems were removed to enable analyses, and the list was verified by 2 of the authors (UM and CL) for accuracy and to ensure validity and prevent bias in the analysis process. Descriptive statistics were used to summarize the heuristic violations and associated severity scores. The severity rating scale from 0 (not a problem) to 4 (usability catastrophe) is shown in Textbox 2.

#### Usability Tests

The usability tests were recorded and transcribed verbatim by a professional transcription company. All transcripts were checked and validated by the first author (UM). Analysis of the video recordings was inspired by program theory development using an inductive approach, taking both verbal and visible conduct into consideration [39]. Transcripts were analyzed thematically using an iterative coding procedure. The focus of the analysis was on the features of the intervention that needed to be redesigned with regard to function, content, and design. Overall, 2 authors (UM and CL) individually read the transcriptions and viewed the video recordings to acquire a comprehensive understanding. The categories were identified using an iterative process of reading and rereading the transcripts. Patterns were searched for, and usability issues were coded into categories. The first coding was initiated by the first author. Next, the coding was presented and discussed between the 2 authors (UM and CL), and boundaries for coding were established jointly [39].

The analysis of the SUS score was conducted according to the scoring strategy of Brooke [40], with the score for each item ranging from 0 to 4. The score from positively worded items (1, 3, 5, 7, and 9) is calculated as the scale position minus 1. For the negatively worded items (2, 4, 6, 8, and 10), the score is calculated as 5 minus the scale position. The sum of the scores is then multiplied by 2.5 to obtain the overall value of SUS ranging from 0 to 100. The average SUS scores were used to identify average satisfaction [40]. According to Bangor et al [38], the average SUS score of approximately 70 can be interpreted as good or acceptable [38].

## Results

### Heuristic Evaluation

**Figure 1:**
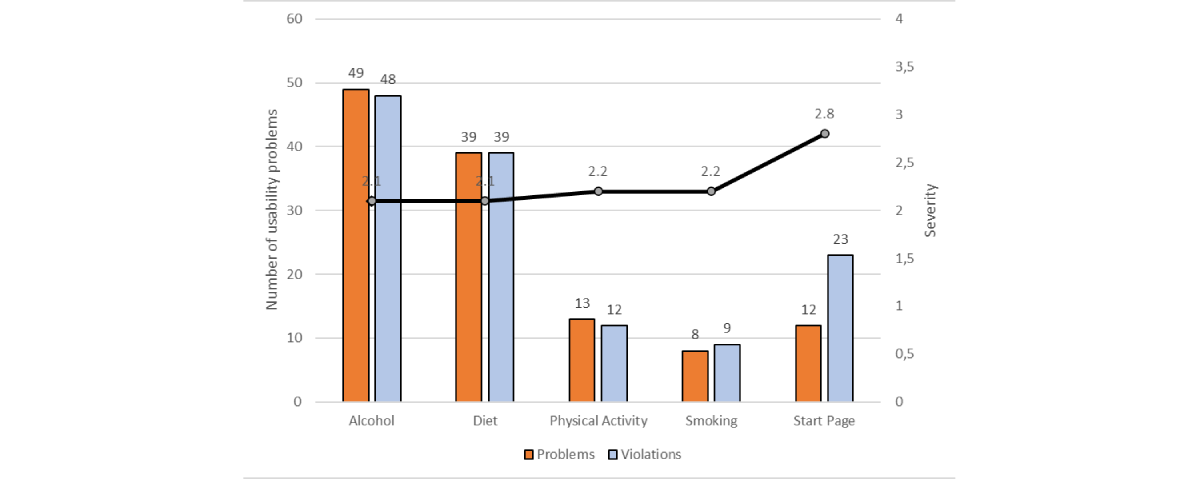
Number of usability problems identified presented by place of occurrence, counts of heuristic violations identified by the 15 participants.

The heuristic evaluation resulted in a total of 121 usability problems and 131 heuristic violations reported by 15 participants. The usability problems identified through heuristic evaluation are summarized and presented by place of occurrence (eg, alcohol, diet, physical activity, and smoking modules as well as the start page), number of heuristic violations, and mean severity ratings in Figure 1. The alcohol module generated the maximum number of usability problems and problem descriptions (n=49), followed by the diet module (n=39), the physical activity module (n=13), and the smoking cessation module (n=8). The start page also had usability problems (n=12). The average severity ratings ranged from 2.1 to 2.8 (on a scale of 0-4). The start page module had the highest severity rating, that is, 2.8.

The line in Figure 1 shows the mean severity ratings for usability problems on a scale of 0 to 4, where 0=none, 1=cosmetic problems, 2=minor problems, 3=major problems, and 4=usability catastrophe.

Of the 10 types of heuristic violations depicted in Table 1, categories 2 (*Match between system and the real world*) and 4 (*Consistency and standards*) dominated at 33 and 32, respectively. The heuristic categories 1 (*Visibility of system status*; n=4) and 10 (*Help and documentation*; n=3) had the lowest violations across all views.

**Table 1:** Presentation of the frequencies of 10 heuristic violations sorted by Nielsen’s heuristics reported by the 15 participants.

Violations	Number of participants
Visibility	4
Match	33
User control	6
Consistency	32
Error	8
Recognition	8
Flexibility	21
Aesthetics	10
Recover	5
Help	3

**Figure 2:**
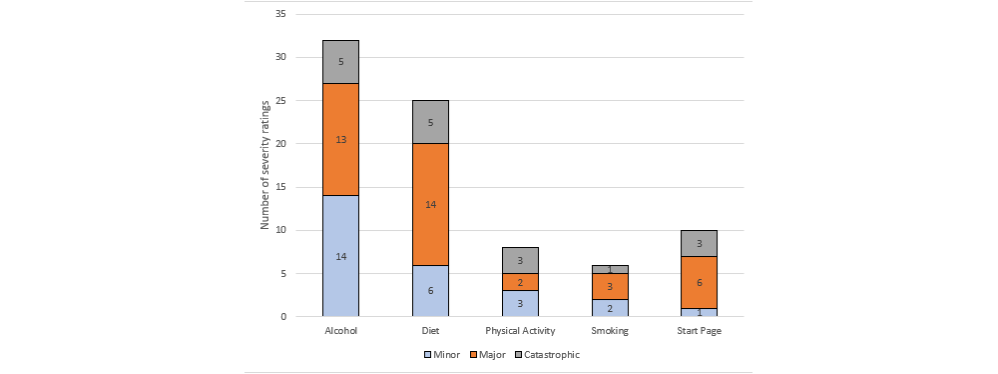
Number of severity ratings (minor, major, and catastrophic) for each module as reported by the 15 participants.

Most severity ratings were in the major severity category, indicating that fixing the problem should be given high priority (severity rating 3). The problems categorized with the highest severity rating were in the alcohol and diet modules. Most of the minor usability problems, that is, fixing those problems should be given low priority (severity rating 2), were also in the alcohol and diet modules. There were also a total of 17 problems reported as usability catastrophes across all modules, indicating the imperative to fix the problem before the product can be released (severity rating 4). Figure 2 shows severity ratings across system views.

When analyzing the nature of usability problems, the heuristic evaluation revealed that major usability problems and catastrophic ratings concerned shortcomings in displaying easy-to-understand information to the users or technical errors. Examples of these types of usability problems provided by participants are as follows:

Navigation unclear. There’s a constant mixture of headings [in the various modules], unclear headlining, different selectable functions, sometimes with hidden text. You don’t know where you are

in the intervention

If the diagram’s x- and y-axes don’t have any labels, you don’t understand because the table headings are unclear and don’t stand alone.

In the figures it’s unclear which direction the scales go in, you don’t understand the colors, it’s confusing.

Several choices of wording, and the concepts are difficult and complicated. Think about having simple, consistent wording and simpler language.

It’s not possible to save all the choices you’ve entered, you can’t change your choices. The planning disappears when you browse through the tabs.

You can’t go back easily. In other words, it’s not possible to find your way “home” easily, there’s no home button.

### Usability Tests

A total of 5 high school students, 3 adolescent girls and 2 adolescent boys, participated in the usability testing. The analysis of the data from the transcripts revealed 3 main categories of barriers that limited usability: (1) design, (2) content, (3) and functionality.

#### Design

*Design* refers to how the intervention is perceived visually. The data showed that the design of the prototype influenced engagement and interest among users, thereby motivating or hindering user engagement with the intervention. Design comprises both aesthetics, that is, how attractive the intervention is perceived to be, and clarity, that is, how the intervention is structured and presented. For example, participants described how clear headlines and appealing infographics were integral to usability. Moreover, participants highlighted the use of clearly presented text, figures, and tables as a way to improve the usability of the intervention. In addition, characteristics of the intervention such as the use of attractive pictures, symbols, and videos were described as further improving usability:

And then I wonder if you could make it a bit more fun or something /.../ to have some background color other than white.

Adolescent 2

Perhaps it would be good to have images? I don’t think it captures the user’s interest much [without images].

Adolescent 1

Regarding the font size, participants preferred a larger font size and suggested clearly defined headlines:

/.../ an intervention that looks a bit half-hearted and where things aren’t, like, centered or whatever. Perhaps it looks less professional than it actually is, and so you don’t trust it as much /.../ because you’ve put so much effort into it being scientifically correct, so you could put a bit of effort into it looking good or, like, fixing it.

Adolescent 4

/.../ and bigger, clearer headings. I think bullet points are good, easy to read /.../ I don’t think you get particularly excited using it.

Adolescent 1

#### Content

The second category refers to both the quality and quantity of intervention *content*. Problems relating to quantity were described as too much text, with large blocks of text making the information difficult to process, which subsequently limits the usability:

/.../ there’s a lot of text. It gets a bit, like, too much text to manage to read through everything. /.../ you don’t want there to be too much text. It should be, like, quite quick and easy.

Adolescent 5

You can quite easily get tired with a lot of text.

Adolescent 3

Sometimes less is more /.../ if it’s simpler, it’s easier.

Adolescent 4

The quality of the content referred to the terminology whereby language was perceived as too heavy, too complicated, and difficult to comprehend. Indeed, words, phrases, and concepts were perceived as unfamiliar and not tailored to the target age group. Thus, engaging with the content was too taxing and limited the usability:

What on earth is “moderate level” and what on earth is “strenuous level”?

Adolescent 4

“Dietary index”? What does “dietary index” mean?

Adolescent 1

Very strange words. People don’t use them at all!

Adolescent 3

#### Functionality

The third category *functionality* referred to the need for the app to be effortless to navigate, to be quick to use, and to have a logical flow, according to participants who wanted an easy-to-use interface. Poor functionality was considered a major usability barrier. Participants described that usability was limited when navigation was complex and included multiple modules or functions. For example, participants stated that scrolling to find information required too much effort and the intervention needed to be easier to grasp:

I think fewer stages would be good.

Adolescent 1

Yes, because otherwise perhaps it’s a bit so-so, that you go in and first you can click there, and then click there /.../ in other words, it gets too much.

Adolescent 2

Participants stated that time was precious and they did not want to spend time navigating unnecessarily, such as entering what they had been eating or how many activity minutes they had participated in. Thus, engagement with the app must be effective and targeted to facilitate usability:

You should be able to get an overview extremely quickly.

Adolescent 4

Like, how many times I ate fruit or berries last week? Yes, I might have eaten fruit once. I might have eaten it ten times, I might not have eaten it at all. I don’t know. It was a bit difficult.

Adolescent 5

Participants also described the importance of a logical flow between different components of the app. In addition, the features that guided or prompted navigation of the intervention could facilitate usability:

You need a bit more help orientating yourself, where you are. Or some kind of main menu that comes up, and then you can tap on alcohol and after you’ve tapped on that subheadings appear. Then you can choose between them.

Adolescent 4

Maybe [it would help if] everything is in categories instead, and you tap, and maybe then it appears. Not showing everything there from the start ... instead, you can go into what you’re interested in.

Adolescent 1

Furthermore, relating to the logical flow within the intervention, there was a desire for consistency between the different modules to improve the usability of the intervention:

Because maybe you can’t have the same subheadings for everything, but that they’re still very consistent, that there are reminders and text messages, then they should be in all [modules] so you can get familiar with it and find things.

Adolescent 1

### The SUS Score

The results of the SUS scores are given in Table 2. As shown in this table, the analysis of the SUS score identified that the intervention was rated with an average score of 66.6. According to Bangor et al [38], an average SUS score below 70 indicates that the system has shortcomings that need to be addressed. Of the 5 participants, one rated the LIFE4YOUth app as poor, 2 as average, and 2 assessed it as good.

**Table 2:** Result of the System Usability Scale.

Questions	P1^a^	P2	P3	P4	P5	Average
1. I think that I would like to use this app	4	4	3	2	3	3.2
2. I found the app unnecessarily complex	1	0	2	1	1	1
3. I found the app easy to use	1	3	3	2	2	2.2
4. I think I would need support from a technical person to use this app	4	4	3	3	4	3.6
5. I found the various functions in this app were well integrated	2	3	4	2	2	2.6
6. I thought there was too much inconsistency in this app	1	2	3	2	2	2
7. I would imagine that most people would learn to use this app very quickly	0	3	4	3	4	2.8
8. I found the app very cumbersome to use	1	3	3	2	4	2.6
9. I felt very confident using the app	1	2	3	1	4	2.2
10. I needed to learn a lot of things before I could start using this app	4	4	3	4	4	3.8
SUS^b^ score (sum×2.5) maximum 100	47.5	70	77.5	55	75	66.6

^a^P1-5: person 1-5.

^b^SUS: System Usability Scale.

## Discussion

### Principal Findings

As described in our previous protocol study for a participatory design [34], the prompt expansion of device capability presents many challenges for developers of mHealth interventions, especially when designing interventions that aim to affect multiple individual lifestyle behaviors [25]. This study investigated the usability of the LIFE4YOUth intervention targeting health behaviors among high school students. Previous studies have suggested using a combination of different usability methods to provide insights to developers about potential usability problems [41]. This paper reports data from the first stage of a formative research process [42], which included heuristic evaluation and usability testing.

The heuristic evaluation revealed that the major usability problems and the catastrophic ratings concerned shortcomings such as information display and comprehensive information, meaning that the intervention needed to speak the users’ language with consistent information that appears in a natural and logical order for the users. The results from the usability testing showed that design (aesthetics and clarity), content (quality and quantity), and functionality (effortless and logic flow) enabled usability. The findings of this study are consistent with those of previous research, which found that participants wanted features that reduced the amount of time and effort required from them [43]. The amount of data participants are expected to enter into self-monitoring apps should be carefully considered in future intervention development [44] because the results showed that frustration with a large quantity of data is one of the most common complaints of users and results in apps being deleted entirely [45].

Engagement was an issue closely related to usability. For instance, participants explained that when the intervention did not appear to have a logical flow, they would quickly cease to use it. Previous research has stressed that engagement refers both to how a user interacts with technology and their emotional response to it [46]. In a study investigating usability barriers and enablers for interventions targeting harmful drinking in young adults, participants stated that positive experiences of usability made them engage more with the intervention and made them more likely to keep using it [43]. In a think-aloud study among adult smokers and drinkers, users revealed their choice of smoking cessation or alcohol reduction apps to be influenced by the apps immediate look and feel, social proof, and titles judged to be realistic and relevant [47,48]. Individuals seem more motivated to engage with and process information more thoroughly if the message is personally relevant and meaningful [49]. Theoretical models of user engagement propose that an individual’s characteristics and personal circumstances may influence their user experiences of digital interventions [50]. Engagement is an ongoing issue for mHealth intervention development. Low login rates and limited use of interventions are issues consistently reported in the literature, and higher engagement through logins and repeated use is associated with better effects of the intervention [51].

According to previous research, the most important factors during the design process are flexibility and responsiveness to the input and feedback of the target audience [52]. Optimizing usability for mHealth interventions is a critical step in the development process. Young adults, the age group with the highest use of mHealth interventions, have high expectations of digital products. If an intervention does not have optimal usability, engagement will be compromised [46]. We agree with previous researchers who conclude that it is not good enough to have an evidence-based intervention per se, but rather, aspects such as delivery, design, aesthetics, usability, and functionality need to be carefully considered [43]. As there is a high demand for interventions that target skilled technology users, such as high school students, the LIFE4YOUth mHealth intervention needs to be iteratively refined and improved and will, thereafter, be pilot tested. An RCT will be conducted to determine the efficacy of the intervention at a later stage. If effective in the subsequent RCT, the program has the potential to be implemented nationally through school health services. According to the World Health Organization, education can play an important role in health promotion of the youth [53]. School multidisciplinary teams provide good accessibility for high school students, and therefore, a natural setting for attempting to endorse health behaviors for as many high school students as possible [15].

### Strengths and Limitations

A limitation of the study is the small and partly nonrepresentative sample that highlights the need for caution when interpreting the results. The results cannot be used for far-reaching conclusions.

Combining heuristic evaluation and usability testing is a strength of this study. Heuristic evaluation provided insights to developers about potential usability problems, particularly in terms of identifying problems with user interface usability. The results from the heuristic evaluation were also used as inspiration to create tasks applied in the usability tests. Usability tests provided knowledge regarding whether specific tasks could be performed in the sequences of actions they were designed for to give direct input into how real users used the system.

The heuristic evaluations were performed wherever the participants preferred, for example, in the participant’s home. This was done to facilitate participation and to optimize that the participants felt no time pressure. Hence, the validity of the data could not be controlled for. This study was not conducted to identify every usability problem with the mHealth intervention but instead to show how heuristic evaluation and usability testing with a small number of users could identify a large proportion of usability problems and assist in making significant improvements to an mHealth intervention targeting multiple health behaviors. The methods used were valuable in identifying not only major areas and themes that needed modification but also smaller, easily fixed problems that users encountered.

### Conclusions

Through participatory design using heuristic evaluation and usability testing, this study resulted in in-depth knowledge regarding the aspects of intervention content and structure that end users (eg, high school students) considered important. This knowledge can be used when designing an mHealth intervention targeting multiple health behaviors. In summary, heuristic evaluation showed that the major usability problems and the catastrophic ratings concerned information display and language use. Usability testing showed that design (aesthetics and clarity), content (quality and quantity), and functionality (effortless and logic flow) enabled usability. This knowledge is valuable in guiding further development of a final version of the novel multiple mHealth intervention program LIFE4YOUth.

## Appendix

Multimedia Appendix 1 Protocol used for participants to report usability problems tied to the place of occurrence, usability problem, problem description, heuristics violated, and severity rating scoring.

## Abbreviations

mHealth: mobile health
RCT: randomized controlled trial
SUS: System Usability Scale
