# Supplementary material for: Development of an Intervention Targeting Multiple Health Behaviors Among High School Students: Participatory Design Study Using Heuristic Evaluation and Usability Testing
Source: JMIR Mhealth Uhealth. 2020 Oct 29;8(10):e17999. doi: 10.2196/17999 (PMC7661261; doi:10.2196/17999)
Supplement: Multimedia Appendix 1 [file mhealth_v8i10e17999_app1.docx]

| Place of occurrence | Usability  problem | Problem description | Heuristics violated | Severity rating |
| --- | --- | --- | --- | --- |
|  |  |  |  |  |
|  |  |  |  |  |
|  |  |  |  |  |
|  |  |  |  |  |
|  |  |  |  |  |
|  |  |  |  |  |

Multimedia Appendix 1. Protocol used for participants to report usability problems tied to the place of occurrence, usability problem, problem description, heuristics violated, and severity rating scoring.
